# Draft Genome Sequences of Six Ruminant *Coxiella burnetii* Isolates of European Origin

**DOI:** 10.1128/genomeA.00285-14

**Published:** 2014-05-15

**Authors:** Karim Sidi-Boumedine, Richard J. Ellis, Gilbert Adam, Myriam Prigent, Øystein Angen, Anna Aspán, Richard Thiéry, Elodie Rousset

**Affiliations:** a ANSES Sophia-Antipolis Laboratory, Sophia-Antipolis, France; b Animal Health and Veterinary Laboratories Agency, AHVLA Weybridge, New Haw, Surrey, United Kingdom; c National Veterinary Institute, Frederiksberg C, Denmark; d National Veterinary Institute of Sweden, Uppsala, Sweden

## Abstract

*Coxiella burnetii* is responsible for Q fever, a worldwide zoonosis attributed to the inhalation of aerosols contaminated by livestock birth products. Six draft genome sequences of European *C. burnetii* isolates from ruminants are presented here. The availability of these genomes will help in understanding the potential host specificity and pathogenicity and in identifying pertinent markers for surveillance and tracing.

## GENOME ANNOUNCEMENT

The intracellular bacterium *Coxiella burnetii* is the causative agent of Q fever, a zoonotic and an abortifacient disease which occurs worldwide. Domestic ruminants are considered to be the main source of human infection with *C. burnetii*. Indeed, it spreads from ruminants to humans via the inhalation of dust and aerosols contaminated by the birth products of livestock (1). Being mainly asymptomatic in humans and animals, the disease poses a challenge to clinicians, thus delaying diagnosis and treatment or prophylaxis.

Numerous knowledge gaps in the understanding of this organism and of Q fever epidemiology (including in domestic ruminant populations) were highlighted in a recent review (2).

We have sequenced six new *C. burnetii* strains isolated from different ruminant hosts (cattle, sheep, and goats) originating from Denmark, France, and Sweden. This corresponds to an increase in the number of available *C. burnetii* genomes originating from the main reservoir of disease (i.e., ruminants).

Each strain was grown in cell culture, and the total genomic DNA was extracted from purified *C. burnetii* and converted to sequencing libraries using the Nextera XT kits (Illumina). These were normalized and pooled before sequencing either on an Illumina GAIIx instrument with 2 × 120 paired-end reads or on an Illumina MiSeq with 2 × 250 paired-end reads. For each isolate, the A5-miseq pipeline (3) was used to perform read trimming and correction, contig assembly, crude scaffolding, misassembly correction, and final scaffolding. The scaffolds were then reordered with Mauve (4) using *C. burnetii* RSA493 (Nine Mile) as the reference genome (accession no. NC_02971). Capillary sequence data were also used to fill in some gaps in the assemblies.

Genome sequencing for the *C. burnetii* isolates Cb_B1, Cb_C2, Cb_O184, EV-Cb_C13, Cb_B18, and EV-Cb_BK10 resulted in high coverage assemblies of the ~2-Mb genomes (between 200- and 400-fold), which should represent most of the functional annotated genes and allow for comparative studies using these genomes. The final number of contigs for each strain varied between 37 (EV-Cb_BK10) and 268 (Cb_O184), with a maximum contig size of between 83,061 and 350,015 bases and *N*_50_ values between 17,735 and 102,549 bases.

Comparisons of the assembled genomes from this study with the completed reference genomes revealed that the majority of contig breaks in our assemblies corresponded to the positions of insertion sequence (IS) elements in the reference genomes. We were also able to identify several regions of insertions, deletions, and rearrangements. The genomes of bovine-origin isolates were most similar to the RSA493 (Nine Mile) reference genome, while the genomes of the ovine and caprine isolates were more divergent. The assemblies also indicated the presence of a single plasmid in each strain.

To provide essential foundations for effective and sustainable control or intervention strategies, it is imperative to address biodiversity, host/environmental niche correlations, and comparative strain investigations of host-microbe interactions, using refined genomic and postgenomic methods. The availability of these six new genome assemblies together with other publicly available sequence data for this species (5–8) will help in performing comparative genomics to understand the genome differences between the *C. burnetii* strains, including rearrangements, insertions, and deletions, thus contributing to the identification of relevant molecular and virulence markers.

### Nucleotide sequence accession numbers.

The assembled genomes of these six *C. burnetii* isolates have been deposited at the European Nucleotide Archive under accession no. CCAH000000000, CCAI000000000, CCAJ000000000, CCAK000000000, CCAL000000000, and CCAM000000000.
